# Do Vibrant Places Promote Active Living? Analyzing Local Vibrancy, Running Activity, and Real Estate Prices in Beijing

**DOI:** 10.3390/ijerph192416382

**Published:** 2022-12-07

**Authors:** Yuan Lai, Jiatong Li, Jiachen Zhang, Lan Yan, Yifeng Liu

**Affiliations:** 1 Department of Urban Planning and Design, School of Architecture, Tsinghua University, Beijing 100084, China; 2 Marron Institute of Urban Management, New York University, New York, NY 10011, USA

**Keywords:** urban vibrancy, physical activity, neighborhood development, urban analytics

## Abstract

Although extensive research has investigated urban vibrancy as a critical indicator for spatial planning, urban design, and economic development, the unclear relationship between local vibrancy and active living needs to be clarified and requires more in-depth analysis. This study localizes urban vibrancy at both hyper-local and neighborhood scales by integrating high-resolution, large-scale, and heterogeneous urban datasets and analyzing interactions among variables representing vibrancy’s environmental, economic, and social aspects. We utilize publicly available urban open data, Points of Interest requested from API, and leisure running trajectories acquired through data mining to investigate the spatial distribution of various vibrancy indicators and how they interact with physical activity at the local scale. Based on these variables, we then construct linear regression models and Geographically Weighted Regression (GWR) models to test and estimate how local vibrancy and physical activity relate to residential real estate characteristics. The results reveal the strong impact of urban form on local vibrancy but not physical activeness. At the neighborhood level, all vibrancy factors are statistically significant to local residential real estate prices but with different interactions based on location. Our study highlights the importance of accounting for locality and different physical, environmental, social, and economic factors when analyzing and interpreting urban vibrancy at a granular scale within a city.

## 1. Introduction

Urban vibrancy is a critical measure of local liveliness for urban planning, real estate investment, and city management. It reflects many aspects of development including technological innovation [1], local economy [2], corporate growth [3], real estate development [4,5], safety [6,7], public health [8], and social cohesion [9]. Like other large cities worldwide, such connotation also applies to Beijing that a vibrant neighborhood usually implies busy traffic, bustling pedestrians, street commerce, diverse activities, and well-designed public space with a sense of place. A vibrant neighborhood may sound attractive for urban development, but does it promote active living? The World Health Organization (WHO) defines an active city as one with improving opportunities and resources in both built and social environments to enable citizens to be physically active [10]. According to the WHO’s latest global status report on physical activity in 2022, people who reach the recommended levels of physical activity have a 20–30% reduced risk of premature death and 7–8% lower risks of cardiovascular disease, depression, and dementia [11]. Although previous studies indicate that vibrancy is directly linked to human activity—especially collective behaviors such as daily commuting [12], leisure activities [13], and socioeconomic activities [14]—it is unclear how vibrancy is related to active living.

Investigating the complex relationship between vibrancy and physical activity provides critical insights for planning healthy and active cities [15]. Unfortunately, there is a limited understanding of such complex relationships, mainly due to three obstacles. First, it is difficult to define, observe, quantify, and analyze local vibrancy with data at a much finer spatial resolution. Such a localized vibrancy measure requires proper quantification, normalization, and standardization. Furthermore, it is challenging to integrate data representing physical activity and analyze their behavioral patterns along with localized vibrancy in a spatial model. Finally, it is necessary to investigate the complex interaction between vibrancy and physical activity within specific socioeconomic contexts, since their intra-city variation can be related to different levels of neighborhood development. In summary, the potential value of the vibrancy measure still needs a complete analytical framework that integrates potential implementation for promoting active living [16].

Regarding the above obstacles, this study uses Beijing as the study area to analyze the relationship between localized vibrancy and physical activity and how they interact with local real estate characteristics. Our research objective is three-fold: First, we aim to identify vibrancy’s data representation, quantitative proxies, spatial–temporal dynamics, and underlying relationships with a wide range of local physical–environmental–social–economic factors. Relevant findings may extend our scientific understanding of such dynamic urban phenomena. We then investigate the relationship between localized vibrancy and physical activity by integrating the outdoor running trajectory data. Finally, we analyze how vibrancy and active living interact within different locations and neighborhoods defined by real estate property characteristics. Such investigations explore the potential utilization of neighborhood vibrancy measures and how to support future planning decision making and operation based on high-resolution vibrancy quantification. 

The rest of the paper proceeds in the following order: The Research Background introduces critical concepts, terminology, methods, and findings based on previous relevant studies. Focusing on Beijing, we describe key questions and hypotheses according to the research framework. The Data and Methods section demonstrates the computation, quantification, and modeling processes for estimating local vibrancy and extracting meaningful patterns from various urban data resources, as well as analyzing the relationship between vibrancy measures and physical activity. The Findings section presents quantification output, analytical results, and the authors’ interpretations of multiple critical insights that reveal the current issues and future development in Beijing. We then elaborate in the Discussion section to highlight the research contribution and potential implementation, especially on how such analytical insights can support future planning and management decisions. Finally, the authors summarize current limitations and future works, with a conclusion highlighting the importance of this research topic.

## 2. Research Background

### 2.1. Quantifying Vibrancy

Although people usually have a general sense of a “vibrant” place, there is no universal definition of urban vibrancy. Jane Jacobs describes vibrancy as a neighborhood’s positive activeness that makes an enjoyable experience, livable space, and community engagement [17,18]. Urban planners refer to vibrancy with lively street life and the number of people during different times of the day at a more granular scale, such as a neighborhood district, a street block, or a place [17,18,19,20]. It is challenging to quantify vibrancy objectively because of a combined effect involving comparative and multifaceted factors. Conventionally, planners assess local vibrancy by overserving the number of people and street activities during different times of the day. Such a data collection process usually relies on manual observation with recording, sketches, or photography documentation during a site visit, or survey data such as a population census or travel surveys [21,22,23]. In recent years, there have increasingly been new data sources for observing vibrancy thanks to rapid development in urban information and communication technology (ICT) and the Internet of Things (IoT). Alternative information sources include but are not limited to cellphone signaling [24,25,26], social media check-ins [27,28,29], public Wi-Fi usage [30], transit card records [31], GPS devices [32], location-based service (LBS) records [12], surveillance videos [33], soundscape monitoring [34], and night-time light images [35,36]. For example, Botta et al. aggregated cellphone signaling data within two months to measure the vibrancy of seven cities in Italy [37]. Yue et al. acquired citywide cellphone signaling data within 24 hr to capture a snapshot of vibrancy at high spatial granularity [26]. At a neighborhood scale, it is feasible to directly track activity patterns with sampled population groups or recruited volunteers through GPS devices. For example, Wu et al. selected a neighborhood in suburban Beijing and measured local vibrancy by extracting 0.5–1% of residents within the study area and recording their activity trajectories through GPS devices [38].

Besides local population, previous findings also reveal that the intra-city spatial variances of vibrancy depend on a combination of the urban form [39,40], building density and diversity [41], land use characteristics [28,42], transit connectivity [43,44], and environment quality [45]. Such multifaceted urban characteristics create challenges for collecting heterogeneous data, integrating metrics, and defining indicators [25,45]. Various modeling techniques, such as spatial autocorrelation and time series analysis, investigate the spatial–temporal patterns of urban vibrancy [29]. The results indicate that urban vibrancy declines with movement away from the city center, reflecting the urban agglomeration effects and Tobler’s First Law of Geography [16,45]. Although such findings provide empirical evidence to support the urban theory, a lack of analysis on locality at a much more granular scale constrains implementing vibrancy measures into actionable decisions at a neighborhood scale [46].

### 2.2. Vibrancy and Active Living

Active living is a lifestyle that integrates physical activity such as leisure walking, running, biking, or recreational sports into daily routines [10]. Quantifying physical activity relies on combined approaches such as surveys, in situ sensing, manual counting, GPS-enabled tracking devices, and focus group interviews [47]. Although most vibrancy studies estimate human activity using human density, mobility, or activity as proxies, such factors are mainly determined by vehicular traffic and transit networks [24,26]. Cellphone signals, geo-tagged social media feeds, or GPS-enabled application usage may reflect non-vehicular mobility and pedestrian activity, but they primarily represent derived-demand travel, not leisure activity [48]. Besides human activity, spatial distribution and the typology of places provide distinctive semantic signatures for understanding surrounding human activities [49]. Points of Interest (PoIs) represent entities surrounding a specific location, including landmarks, restaurants, coffee shops, bars, or grocery stores [50]. The density and diversity of PoIs are highly related to local land use types and diversity [51]. Such data are usually collected by location-based service operators, such as digital maps, navigation applications, and location-based social networks [52]. A recent study in Shanghai indicated that certain PoI types may reveal a city’s “third place” where people spend time for leisure, social, and recreational activities besides the home and the workplace [53]. Although PoIs do not directly indicate vibrancy, they provide contextual information for interpreting observed vibrancy. Based on such assumptions, Chen T. et al. utilized georeferenced social media posts and PoI data as digital traces of human activity to identify urban vibrancy and its relationship with the surrounding spatial configuration in Hong Kong [54].

Researchers also question whether vibrancy promotes active living and suspect that some vibrancy factors may not always promote physical activity. For example, urban density is a common factor that contributes to local vibrancy [55], but Forsyth et al. discovered that urban density is associated with walking purpose (travel vs. leisure), not the amount of physical activity [56]. Furthermore, directly inferring neighborhood vibrancy from population density or human activity intensity can be problematic due to a combined effect of diverse local physical, environmental, and socioeconomic contexts. Previous findings reveal complex interactions between local vibrancy and surrounding physical, demographic, social, and economic factors, such as the street block size, building density, and demographic diversity [57]. For instance, Lai and Kontokosta demonstrated that integrating and computing variables may explain unique urban contexts and situations at a place for assessing local pedestrian activities [58]. Therefore, a proper interpretation of vibrancy requires integrating other urban metrics to gain more contextual knowledge of the locality and specific neighborhood characteristics.

### 2.3. Vibrancy, Active Living, and Real Estate Development

Previous research proves that local vibrancy is critical for analyzing the real estate market, neighborhood policy making, and investment strategy [59,60,61]. Since neighborhood vibrancy correlates with local environmental, demographic, and social characteristics, it provides a proxy for business development, social integration, streetscape quality, or public safety [2,7,8,9]. Especially for a residential area, vibrancy assessment may guide future economic growth strategies, urban design interventions to promote a more active lifestyle, quality of life improvement, and real estate development. In this aspect, Barreca et al. developed a neighborhood service index as a proxy of local vibrancy to prove the spatial autocorrelation between local housing prices and vibrancy in Turin, Italy [4]. Such underlying associations between pedestrian traffic, property values, and local vibrancy provide unique insights for real estate market development and neighborhood regeneration planning [5,62]. Furthermore, varying neighborhood vibrancy reflects different community development levels and disparity in resource allocation or quality of life [2]. Such local variations reveal the synergetic effects between people, activities, place making, and value creation within a specific physical and social context [63].

Regarding active living, previous findings proved a positive correlation between local housing prices and walkability [64,65,66], representing the quality of space but not actual physical activity. Other studies focused on the driver of activity behavior, and the results indicate the higher income and education levels associated with active living [67,68]. However, few studies investigated how local vibrancy and physical activity are spatially coupled and collectively contribute to local real estate prices.

### 2.4. Vibrancy in Beijing

Beijing is China’s capital city with a land area of 16410.54 square kilometers and approximately 21.893 million citizens [69]. It has a GDP of RMB 3.6T (USD 537.3B) as the national political, economic, educational, and cultural center [69]. The city has a monocentric urban form with distinctive neighborhood types shaped by various planning ideologies through different eras. Its modern era of urban growth expands outwards through a concentric ring road system. Previous studies investigated Beijing’s vibrancy using different data sources such as mobile phone signals, social media check-ins, and GPS trajectories [2,38,70]. Jia et al. quantified busyness and diversity as two vibrancy measures and found high busyness and diversity between the second to the third ring, high busyness between the second and fourth rings, and low busyness and diversity outside of the fifth ring [2]. Wang et al. found similar patterns with higher vibrancy in the second to fifth ring road throughout time and consistent lower vibrancy in the second ring road and outside the fifth ring road [70]. Another study further reveals how street types (i.e., commercial, cultural, residential, mixed, and ecological) may influence demographic vibrancy (e.g., population density, travel behaviors, and pedestrian flows); commercial vibrancy (e.g., the density of stores, social media popularity, market share of local commerce); cultural vibrancy (e.g., density and diversity of cultural activities, public attention, and social influence); and industry vibrancy (e.g., presence of high-tech industries, creative industry, service-based industry, intellectual properties) [71]. Not surprisingly, findings show higher vibrancy on commercial and residential streets and lower vibrancy on cultural and mixed-use streets that are relatively distant from the city center.

Relevant findings also indicate significant relationships between Beijing’s vibrancy and local characteristics. Building density positively correlates with vibrancy (coefficient = 1.25, 10% level significance), and the impact may vary with different levels of urbanization [72]. Transit, especially access to the subway network, also positively impacts vibrancy. Wang et al. estimated different transit accessibility measures and related correlation coefficients to local vibrancy (i.e., proximity to bus stops = 0.02, number of bus stops = 0.02, proximity to subway stations = 0.04, number of subway stations = 0.08) [71]. Even though urban density and transit accessibility often couple with local development, there are inconsistent findings regarding the impact of Points of Interest (POIs). While most studies conclude a positive impact of PoI density, inconsistent findings indicate positive or negative impacts of PoI diversity on local vibrancy [38,70,72]. Such impacts also vary by PoI types, representing the local population, commercial, cultural, and industrial factors collectively driving vibrancy [71].

## 3. Research Framework

This study aims to investigate two primary hypotheses. Since diverse definitions of urban vibrancy indicate the inconsistency of vibrancy measures, this study tests underlying correlations among different aspects of vibrancy, such as population density, business development, or facilities distributions. Thus, we define the following hypothesis to investigate how physical activity relates to other vibrancy factors:

**H1:**  *People who live and work in “vibrant” places tend to be more physically active*.

The second hypothesis assumes neighborhood vibrancy as a co-product of urban geography and a sense of place, obeys the general geographical law of human settlement, and demonstrates anomalies and variations due to place-specific attributes. While Beijing’s vibrancy generally follows a monocentric urban form, the local variance of vibrancy may be associated with real estate price, revealing spatial heterogeneity and neighborhood disparity in environmental, economic, and social characteristics.

**H2:**  *Local vibrancy and physical activity correlate to surrounding housing price that reflects real estate development and quality of life in this neighborhood*.

**Figure 1:**
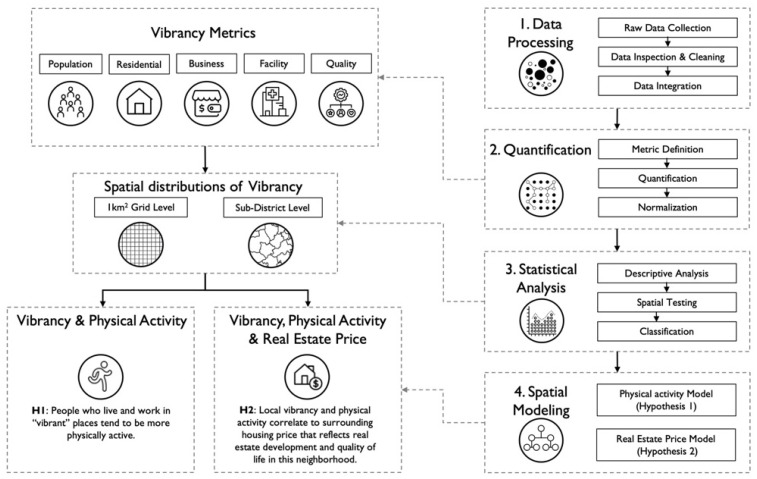
The research framework for this study.

Figure 1 illustrates the overall research framework with four major steps. First, we collect and process diverse data relevant to Beijing’s urban vibrancy. After localizing and integrating different metrics based on a spatial grid, we define and quantify multiple vibrancy metrics based on previous literature and data availability. Furthermore, vibrancy metrics enable descriptive analysis and statistical tests to study the distribution, spatial pattern, composition, and inter-correlations among different factors. Finally, we construct two models to investigate the above hypotheses on the relationship between urban vibrancy, physical activity, and local real estate development.

## 4. Data and Methods

### 4.1. Data Collection and Integration

We used diverse approaches to acquire data from heterogeneous sources, including public data from city open-data portals, data from API requests, and data scraped from websites. Specifically, we identified land use information by collecting data from OpenStreetMap (OSM), which reports street networks, waterways, green spaces, and various land uses. We collected basic demographic information from the 7th National Population Census on sub-district (*n* = 167) total population and density (i.e., the total population divided by land area). To estimate population at a more granular scale, we collected one day’s citywide cellphone utilization data from Baidu Maps as a snapshot of local population distribution. This dataset derives from locational-based services (LBS) that report the origin and destination of daily commutes at a 1 km-by-1 km spatial resolution. We can estimate (1) how many people live and work within each grid cell and (2) the average distance including both outbound commute (live here and travel to work) and inbound commute (live elsewhere and travel here to work). For public resources and facilities, we collected multiple datasets from the Beijing City Open Data Platform (For more information, visit: https://data.beijing.gov.cn, accessed on 15 August 2022), which collectively reports the allocation of community facilities and public amenities such as sports centers, schools, hospitals, libraries, and community service centers. For local commerce and services, we collected Points of Interest (POIs) within the study area via the API provided by Gaode Maps, one of the most popular navigation platforms for residents and tourists.

To estimate physical activity, we collected outdoor running trajectory data from Keep, one of the most popular personal fitness smartphone applications in China. This platform reports anonymized individual jogging activity, including start time, end time, and geographical trajectory. We acquired one month of data using a web scraping technique to collect all documented activity records within the sixth ring of Beijing from 18 July to 18 August 2022. Finally, we collected information on Beijing’s residential apartment compounds from MapTable, a third-party data broker that collects residential property data from online real estate listings (For more information, visit: https://maptable.com, accessed on 15 August 2022). This dataset reports critical information about most residential apartment compounds in June 2022, including geo-location (latitude and longitude), street address, neighborhood district, number of buildings, number of units, average listed price (RMB/sq. m.), built year, and monthly service fee (RMB/sq. m.). All the above datasets can integrate and localize to generate various metrics representing physical, demographic, economic, and social factors (Table 1).

### 4.2. Quantification

**Figure 2:**
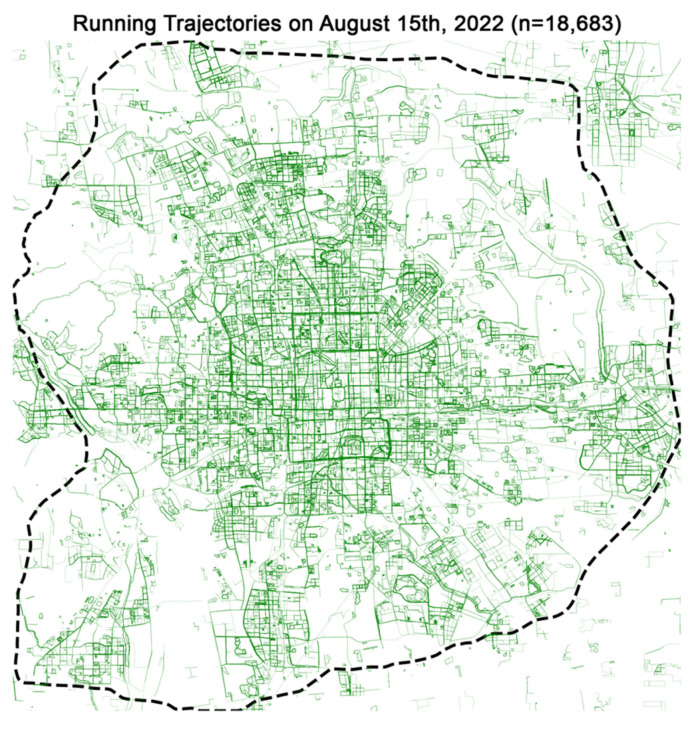
Visualization of extracted daily running trajectories (*n* = 18,683) data within Beijing’s sixth ring road on 15 August 2022.

To integrate heterogeneous datasets, we set a spatial grid with 1 km-by-1 km cells (*n* = 9251) within the sixth ring road of Beijing (Figure 2). Since datasets have various spatial units, including points (e.g., PoI or community facilities), polygons (e.g., green space and water body), and polylines (e.g., street network), we built a spatial processing pipeline in a Python environment to generate measures based on spatial join or proximity search by nearest neighbors. Table 2 summarizes the definition and quantification of different vibrancy metrics representing the base population, proportion of the residential population, local business, public service, and public space quality at the neighborhood scale (Table 2).

We analyzed the physical activity trajectory data following three steps. First, we inspected the raw data by calculating the total length for each trajectory and eliminating unusual activities with zero length (Figure 2). We then extracted information reflecting the activity time by identifying the start and end timestamps and calculating the duration. The division of total length by duration provides an estimated average speed. Finally, we identified the grid cells for each trajectory based on spatial join and specific time period for each segment by hours. For example, if we observe a circular running trajectory that passes a grid cell during hours *t* and *t* + 1, we count for this cell twice during two different hours. Such an iterative computing process enables us to generate estimated hourly activeness after processing all trajectories.

Besides activity trajectory data, we used appropriate spatial join methods for integrating various data sources reflecting the local environment, population, economy, facility, and space quality. To estimate local business, we first identified PoI within each cell and then calculated the total number and distinctive PoI types to represent local business density and completeness, respectively. For business diversity, we quantified the mixture of PoI using an entropy measure within each cell. Since community facilities are more spatially sparse than PoI, we developed a distance measure to estimate the proximity to various facility types (i.e., education, cultural, health, sports, grocery) from each cell. Such a combination of measures reflects accessibility to public amenities and facility resources at different locations. Considering that streetscapes and public space are important drivers of local activity, we calculated the percentage of greenspace and average street quality within each cell based on OSM data and a dataset published by a previous study on Beijing’s streetscape quality [73].

### 4.3. Modeling

The quantification process generates multiple metrics that collectively represent local vibrancy. Statistical summary and spatial data visualization can provide initial insights on distribution, temporal regularity, and geographical patterns. After the initial exploratory data analysis (EDA), we constructed a multivariate model to analyze the composition of neighborhood vibrancy and how different locational, demographic, environmental, socioeconomic, and behavioral factors collectively drive vibrancy (Equation (1)).

(1) $$Activity_{i}=\beta_{1}Population_{i}+\beta_{2}Residential_{i}+\beta_{3}Business_{i}+\beta_{4}Facility_{i}+\beta_{5}Quality_{i}$$

where *i* indicates a specific grid cell, $Activity_{i}$ represents aggregated physical activity based on running trajectories that passed through *i. Population*, *Residential*, *Business*, *Facility*, and *Quality* are independent variables defined in Table 2, and β represents the correlation coefficient for each variable accordingly. After analyzing the composition of vibrancy, we investigated the spatial autocorrelation and temporal integration through spatial testing and a time series analysis. Building upon the simple model described in Equation (1), we further constructed a Geographically Weighted Regression (GWR) model to investigate the underlying interaction among these variables at different geographical locations. We expect that this model can further reveal granular variations and anomalies of factors’ interaction according to the overall GWR model’s performance and estimated local coefficients (Equation (2)).

(2) $$Activity_{i}=\beta_{1\left(u_{i},v_{i}\right)}Population_{i}+\beta_{2\left(u_{i},v_{i}\right)}Residential_{i}+\beta_{3\left(u_{i},v_{i}\right)}Business_{i}+\beta_{4\left(u_{i},v_{i}\right)}Facility_{i}+\beta_{5\left(u_{i},v_{i}\right)}Quality_{i}$$

where $\left(u_{i},v_{i}\right)$ represents the centroid points of the local area based on the geography-based kernel function with an adaptive bandwidth, so the coefficients are location-specific in this model.

Utilizing the localized vibrancy indicators, we further conducted a benchmarking analysis by integrating real estate properties and their proximate vibrancy measures within the sixth ring road in Beijing. A benchmarking analysis compares one entity’s attributes or information against another. It is a common analytical approach for data-driven decision support in real estate financing, building performance evaluation, and urban planning. A typical benchmarking analysis requires quantification, standardization, comparison, and visualization to offer actionable insights on future strategy and decisions to improve performance in areas of concern [74,75]. Specifically, the benchmarking analysis in our study proceeded with the following three steps. First, we spatially joined residential apartment compounds (*n* = 6678) with the grid to estimate local vibrancy measures for each real estate property. Such data integration allowed us to analyze the underlying relationship between real estate price and vibrancy measures. Furthermore, we ran a GWR model to check the statistical significance and coefficient of vibrancy measures on property prices (Equation (3)). The underlying global effects and local variations may indicate how multiple aspects of vibrancy and active living may drive local real estate prices differently depending on specific locations. Finally, we calculated the sub-district level-aggregate measures and constructed a new model for estimating the potential explanatory power of neighborhood vibrancy. This part of the analysis concluded with a descriptive summary of the statistical distribution and spatial patterns of the above measures and model performance metrics.

(3) $$\begin{array}{l} {\mathrm{Price}}_{i}=\beta_{1\left(u_{i},v_{i}\right)}{\mathrm{Building}\_\mathrm{Age}}_{i}+\beta_{2\left(u_{i},v_{i}\right)}{\mathrm{Total}\_\mathrm{Units}}_{i}+\beta_{3\left(u_{i},v_{i}\right)}{\mathrm{Distance}}_{i}\\ \;\;\;\;\;\;+\beta_{4\left(u_{i},v_{i}\right)}{\mathrm{Commute}\_\mathrm{Work}}_{i}+\beta_{5\left(u_{i},v_{i}\right)}{\mathrm{Commute}}_{\_}{\mathrm{Home}}_{i}\\ \;\;\;\;\;\;+\beta_{6\left(u_{i},v_{i}\right)}{\mathrm{Population}}_{i}+\beta_{7\left(u_{i},v_{i}\right)}{\mathrm{Residential}}_{i}\\ \;\;\;\;\;\;+\beta_{8\left(u_{i},v_{i}\right)}{\mathrm{Business}}_{i}+\beta_{9\left(u_{i},v_{i}\right)}{\mathrm{Facility}}_{i}+\beta_{10\left(u_{i},v_{i}\right)}{\mathrm{Quality}}_{i}\\ \;\;\;\;\;\;+\beta_{11\left(u_{i},v_{i}\right)}{\mathrm{Activity}}_{i}\mathrm{II} \end{array}$$

where, for a residential apartment compound *i*, *Price* is its average price (RMB/square meters), *Building_Age* is the number of years since its construction, *Total_Units* is its number of apartment units, *Distance* is the distance to the city center, *Commute_Work* and *Commute_Home* represent the average commute distance for people who live or work here accordingly, and the rest of the variables are defined in the same way as Equations (1) and (2).

Based on the above analytical pipeline and interpretation of the model results, we finally identified critical use-case scenarios with the vibrancy analysis. Identifying potential implementations requires considering future planning decision support in two ways, including both top-down city-scale spatial-planning strategies and bottom-up participatory planning at the neighborhood scale. After synthesizing the urban context of Beijing and our research findings, we identified critical recommendations for future urban decision-making scenarios: (1) resource allocation for future community facilities; (2) spatial planning for promoting physical activities; (3) economic planning for future community business.

## 5. Findings

### 5.1. Localized Vibrancy and Activity

**Figure 3:**
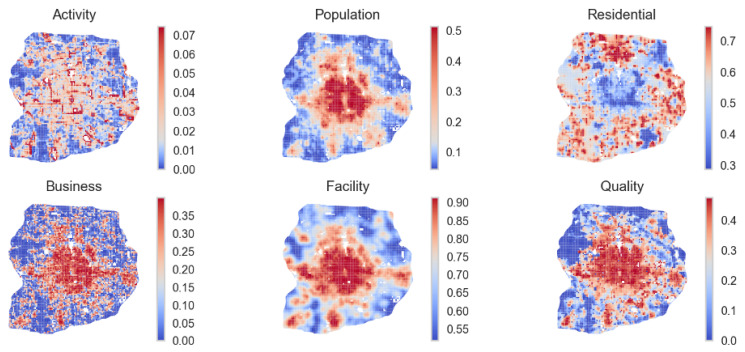
Localized physical activity and vibrancy estimation based on 1 square kilometer grid.

The spatial distribution of vibrancy measures indicates the impact of the monocentric urban form of Beijing and resonates with previous research findings (Figure 3). The correlation matrix of normalized metrics reveals underlying interactions among different aspects of vibrancy. The results show that most metrics have a positive correlation coefficient with each other, indicating that they have synergy rather than conflicts when driving local vibrancy. Such results resonate with Jacob’s observation of urban vibrancy that different elements compose local vibrancy and conduct the “daily ballet” on the streets [17]. Locations with high residential concentration are not “vibrant” based on vibrancy metrics, so we may need to separate the residential and non-residential areas in a city when interpreting vibrancy. Correlation coefficients among vibrancy indicators show that community facilities (educational, cultural, healthcare, sports, and grocery facilities) and public space quality (streetscape quality and green space) are mostly correlated (coefficient = 0.7). Global Moran’s I statistics indicate that these indicators are not distributed randomly but with different levels of spatial autocorrelation, reflecting Tobler’s First Law of Geography. The spatial statistics show that all vibrancy indicators are spatially correlated (*p*-values < 0.0) with a different positive Moran’s I value. Precisely, different levels of spatial autocorrelation follow the order of community facility (0.973), population (0.950), residential (0.818), public space quality (0.811), and local business (0.612). The population and community facility indicators have a Moran’s I value close to 1, indicating the strong spatial clustering effects of these two factors in Beijing. In contrast, Moran’s I value for localized physical activity is 0.407, indicating that physical activeness is not randomly dispersed but without a strong clustering effect compared to vibrancy. 

**Figure 4:**
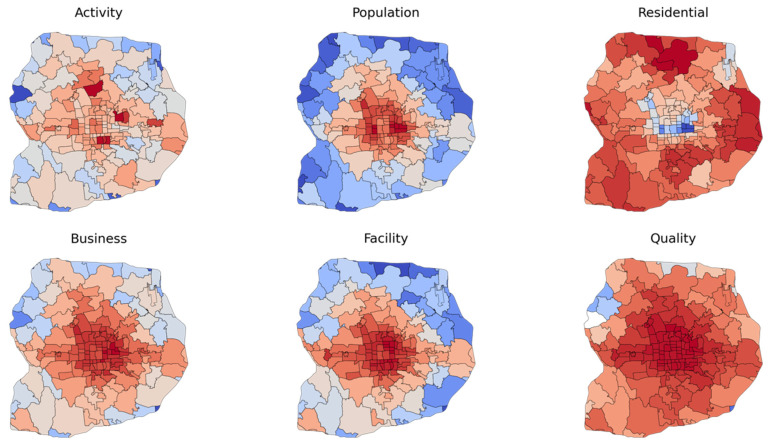
Aggregated physical activity and vibrancy measures at the sub-district level.

We also aggregate vibrancy measures at the sub-district level, which is a common spatial unit that defines a neighborhood for planning decision making and surveys in Chinese cities. The results reveal a general spatial pattern of monocentric urban form and multiple structural effects caused by land use planning (Figure 4). Specifically, population (including residents and workers), business, and facility indicators gradually decrease from the city center, reflecting the agglomeration effect of the urban population, economic activity, and public resources. Comparing the spatial dispersion of business and facility (public-owned amenities) shows that private-led businesses estimated by PoI are more dispersed than community facilities, especially for the northern outskirt sub-districts with a concentration of residential areas. The comparison between population and residential level shows the imbalanced distribution between living and working places. Regarding local physical activity, the results do not show a monocentric spatial pattern based on urban form compared to business or facilities. The hot spots with intense activity are related to city parks. Finally, public space quality’s local coefficients have a graduate spatial gradience without patterns, indicating the spatial disparity of streetscape quality and access to green space. 

### 5.2. Relationship between Vibrancy and Physical Activity

The results from a GWR model reveal how different indicators collectively drive local vibrancy and their interactions based on geography. As Table 3 shows, the GWR model performs better than a regular multivariate regression model according to *R*-squared values and Akaike Information Criterion (AIC). While the global regression model’s results indicate how base population, community facility, and public space quality collectively drive local physical activity, the GWR model reveals the local variation of each variable’s estimated coefficient. The optimal bandwidth for GWR spatial kernel is 720 m, indicating that vibrancy factors may interact differently depending on specific locations. Both models’ results show no consistent effect of local business (measured by PoI) on physical activity, indicating no strong spatial correlation between these two vibrancy factors. A simple GWR linear model further proves that although there is a positive correlation (*p*-value = 0.000), local business has little explanatory power for estimating physical activity (adjusted *R*-squared value = 0.026). Such results indicate that local business as a common measure for vibrancy does not correlate to physical activity.

**Figure 5:**
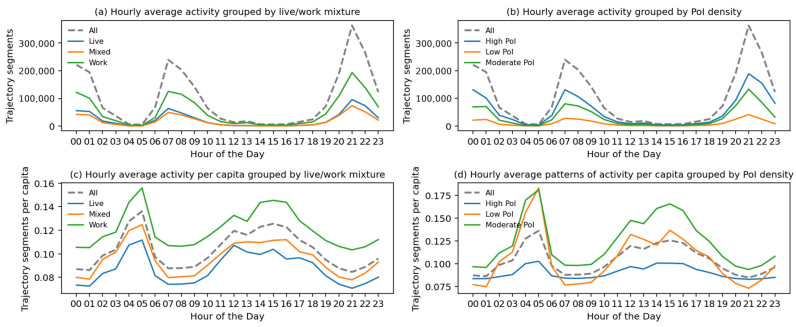
Time series of average hourly patterns of physical activity grouped by live/work mix and PoI density.

In addition to regression models, a time series analysis of hourly aggregated physical activity grouped by local population mixture (e.g., live, work, or mixed) further reveals that physical activity is primarily driven by the local working population, especially at noon (Figure 5). Even though most facility metrics have a correlation coefficient above 0.5, it is uncertain whether they tend to share the proximate location or are distributed evenly with a similar distance from each cell based on such correlation.

### 5.3. Real Estate Property

**Figure 6:**
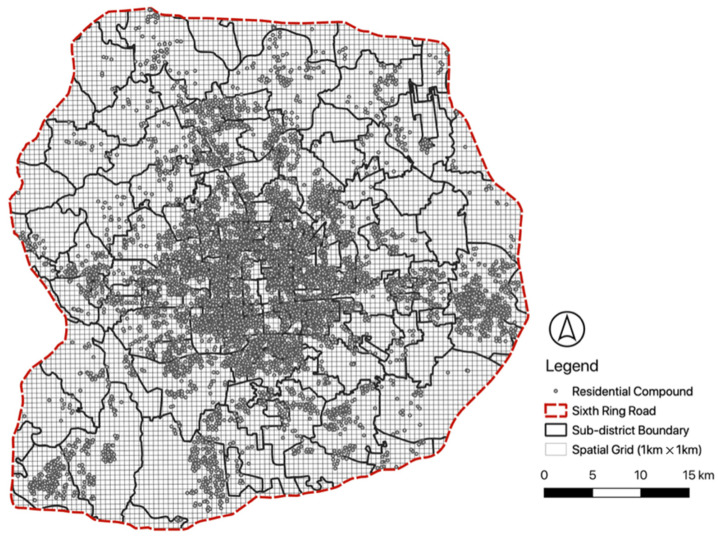
Real estate compounds (*n* = 6678) within sixth ring road in Beijing with the spatial grid (*n* = 9251) and sub-districts (*n* = 167).

The final dataset includes a total of 6678 residential apartment compounds within the sixth ring roads of Beijing (Figure 6). The spatial distribution of these properties reflects rapid real estate development in the last three decades. From the 1990s to the 2000s, the city experienced the highest annual expansion rate (58.91 sq. km. per year) and rapid urban sprawl along with fast-growing private vehicle ownership and the expansion of the subway, light rail, highways, and the ring road system [76,77,78]. During this period, most new neighborhoods were shaped by real estate development dominated by large-scale gated residential apartment compounds within the city [79]. At the same time, new neighborhoods emerged on the city’s outskirts, representing a suburban lifestyle with luxury townhouses and villas [80]. An integrated model with residential apartment compounds’ information reveals the current spatial distribution of real estate properties and their local characteristics (Table 4). All selected independent variables are statistically significant at various levels, but the model’s *R*-squared value indicates a relatively poor overall fit (*R*-squared value = 0.595). The global estimated coefficients also show counterintuitive results, such as the building age (coefficient = 0.045, *p*-value < 0.0001) and public space quality (coefficient = −0.022, *p*-value < 0.1). The GWR model performs better than a regular regression model according to the *R*-squared values and AIC scores. The estimated local coefficients show the spatial heterogeneity of different factors related to local residential apartment prices.

**Figure 7:**
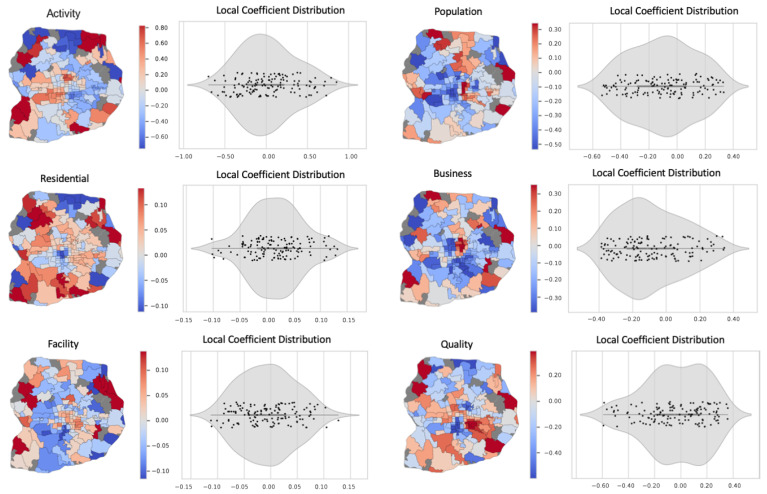
Local coefficient distribution and spatial patterns for vibrancy indicators and physical activity at neighborhood scale.

Visualizing each variable’s local coefficients reveals how these factors collectively correlate to real estate price with geographical variations (Figure 7). Similar to the vibrancy model, the results indicate substantial spatial heterogeneity and local effects that a global regression model fails to capture. Although real estate pricing is a much more dynamic factor beyond the scope of our model, the results still show nontrivial findings that are worth further investigation. Specifically, when looking at the inner areas close to the city center, the results show distinctive coefficients between west and east sub-districts for the local population and business vibrancy estimated by PoI.

## 6. Discussion

Our findings resonate with previous literature and contribute to a more profound understanding of urban vibrancy, mainly in three aspects. Methodologically speaking, they set a conceptual model for understanding the composition of urban vibrancy with local variations and construct an analytical pipeline for processing, integrating, normalizing, and analyzing multiple vibrancy factors. Using Beijing as an example, we demonstrated the computational procedures for data mining and indicator quantification. Furthermore, we utilized new spatial-temporal trajectory data for estimating local physical activity that is fundamentally distinctive to population density or general mobility patterns. This novel data integration and analytics reveal the spatial–temporal patterns of physical activity driven by an active lifestyle rather than a commute. Overall, such quantification can further support data-driven solutions and scientific understandings of the relationship between urban vibrancy and physical activity at a hyper-local scale.

Theoretically, this study reveals urban vibrancy as a complex indicator that can be decomposed into aspects reflecting local population composition, spatial layout, business development, and public facilities. While each vibrancy measure is spatially continuous and autocorrelated based on the First Law of Geography, different types of vibrancy do not always share a positive correlation but form various combinations based on location. This finding indicates that any single aspect indicator (e.g., based on population mobility or PoI density) can be biased and cannot represent the entire spectrum of vibrancy. In particular, our study reveals multiple underlying drivers for local physical activity, including local population size and composition, community facility, and public space quality, but not local businesses. This finding offers critical discussion on balancing street commerce and active living. We suspect that an underlying threshold of business vibrancy and over-developed business vibrancy may negatively impact a sense of community for safe and comfortable outdoor physical activity.

**Figure 8:**
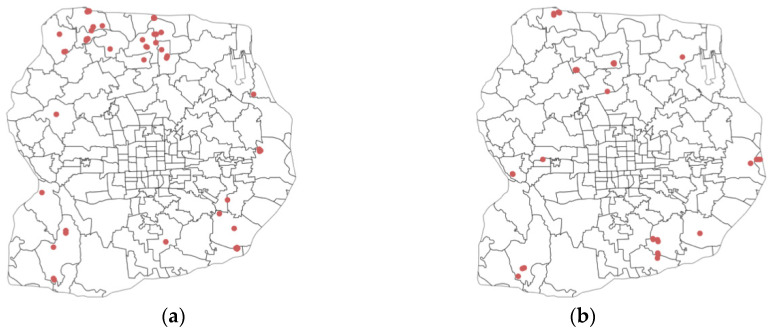
Querying residential apartment compounds with mismatched vibrancy characteristics. (**a**) Most residential with the lowest facility; (**b**) highest activeness with the lowest space quality.

Practically speaking, this study provides potential implementation cases with an analytical pipeline to support data-driven decision making in multiple scenarios. Integrating heterogenous data sources into an analytical pipeline enables generating hyper-local urban contextual awareness for analytics and decision support [81]. For example, descriptive measures on multiple aspects of urban vibrancy allow direct querying and sorting based on different measures at a hyper-local scale. Such analytics can support planners in identifying places with imbalanced vibrancy characteristics or mismatched facility distribution. In this case, a city planner can identify specific residential compounds by asking, “where are the places with the most residential proportion but lowest community facility” (Figure 8a) or “where are the places with most physical activity but lowest public space quality”? (Figure 8b). Such information can inform decision makers about underlying vibrancy issues and guide further on-site investigation of specific neighborhoods.

Despite the comprehensive data collection and analysis, this study still has several limitations in terms of data sources, models, and validation. First, more data sources are needed to represent the multifaceted nature of urban vibrancy, especially the digital representation of people’s physical activities, social behavior, and public opinions. Even though Keep is the most popular application in China, there is still a data representation bias toward the users of this smartphone application. Moreover, our current analysis cannot capture the informal business, facilities, or built areas if they are not yet digitized as data. This raises the question of the disparity of local digital representation, which may require a hybrid approach integrating big data analytics with conventional site surveys for ground-truth observation and inspection [82]. Due to our research interest, this study did not integrate social media data that may reflect underlying social networks, public attitudes, or collective sentiment on the local scale. One of our previous studies in New York City revealed that social media text data analytics may provide a proxy for estimating the dynamic change of attitude and sentiment as an early sign of neighborhood change [49]. Secondly, since our research aims to provide analytical insights for spatial decision making, the current models mainly focus on the spatial distribution of urban vibrancy but less on the temporal changes in local vibrancy. Therefore, we have not yet further investigated how locality changes during different times throughout the day. Such limitations may constrain the understanding of night-time vibrancy, which plays an increasingly important role in local street life and the neighborhood economy. Finally, since vibrancy measures are multi-dimensional with spatial heterogeneity, it is challenging to validate estimated local vibrancy with another singular data source.

Regarding the above limitations, in future work, we plan to integrate more datasets from new sources such as social media, purchasing records, or public opinions collected through crowdsourcing for quantifying people’s behavior and sentiment that may reveal local cyber-social dynamics. We expect such data integration to capture more socioeconomic factors, so we can investigate how vibrancy can be culturally specific for different neighborhoods or cities. We also plan to continue analyzing the vibrancy data with further time series analysis to unpack its temporal patterns citywide and locally. Finally, the research team plans to collect first-hand observation data and validate computational results with the ground truth observed during the site survey. We expect that such a manual process may provide more qualitative information for understanding local contextual and situational factors relevant to urban vibrancy.

## 7. Conclusions

This study investigated the spatial–temporal dynamics of urban vibrancy in Beijing and how various factors correlate with neighborhood vibrancy at a hyper-local scale. The research findings prove the strong spatial autocorrelation of vibrancy measures that resonate with the planning theory of urban form and the First Law of Geography, but not all vibrancy measures are positively correlated. Although physical activity is related to the local population, residential percentage, business, and public facilities, it does not show a strong spatial gradient that matches the monocentric urban form as vibrancy. GWR models perform better than regular regression models for both hyper-local and neighborhood (sub-district) scales, indicating the importance of locality for analyzing vibrancy. For real estate analysis, the GWR model explains about 82% of the local vibrancy variation and reveals different interactions among vibrancy factors according to the spatial distribution of coefficients at the sub-district level. In conclusion, such integrated high-resolution data reveal local variations of urban vibrancy and provide more actionable insights for future neighborhood development involving spatial planning, human mobility, facility allocation, and many other factors related to urban vibrancy.

## Figures and Tables

**Table 1:** Data collection.

Name	Description	Spatial Unit	Sample Size	Source	Acquisition Approach
Population	Total population within each sub-district	Sub-district	167	Census Data	Download
Real Estate Properties	Built year, total units, average price	Geo-location	6678	MapTable	Download
Streets	Street segments with walking quality score	Polyline	45,090	Beijing City Lab	Download
Green Space	Land parcel representing parks and green open space	Polygon	5045	OpenStreetMap	API request
Facilities	Location of community facility and public amenities	Geo-location	14,171	Beijing Open Data	Download
PoIs	Points of Interest	Geo-location	180,594	Gaode Map	API request
Physical Activity	Outdoor running trajectories	Polyline	660,891	Keep	Web scraping
Live–Work Pattern	Local live and work population composition and average commute distance	Grid Cell (1 km)	6927	Baidu	API request

**Table 2:** Neighborhood vibrancy metrics and indicators.

Indicator	Metric	Definition
Population	Population density	Estimation of the total living and working population
	Inbound distance	Average commute distance from home to the cell (work)
	Outbound distance	Average commute distance from the cell (home) to work
Residential	Local residents	Proportion of residents among total local population
Business	PoI density	Number of PoIs within the cell
	PoI diversity	An entropy measure indicating the diversity of PoIs (0–1)
	PoI completeness	A percentage measure indicates the completeness of PoI categories
Facility	Education	A proximity measure for estimating distance to kindergarten, elementary school, and high school
	Cultural	A proximity measure for estimating distance to community cultural centers, performance centers, and libraries
	Health	A proximity measure for estimating distance to community health care centers and senior care centers
	Sports	A proximity measure for estimating distance to sports facilities, youth sports and recreation centers
	Grocery	A proximity measure for estimating distance to local food market and grocery stores
Quality	Streetscape quality	An index score for street quality (0–100)
	Green space	Percentage of green space within the cell

**Table 3:** Results from a global regression and GWR model.

	Global Regression		Geographically Weighted Regression		
Independent Variable	Coeff.	Std. Err.	Mean	Min	Max
Vibrancy-Population ***	−0.111	0.019	−0.288	−15.961	18.422
Vibrancy-Residential ***	−0.052	0.011	−0.047	−7.237	4.314
Vibrancy-Business	−0.019	0.015	−0.000	−2.892	4.994
Vibrancy-Facility ***	0.165	0.017	−0.012	−42.557	15.328
Vibrancy-Quality ***	0.230	0.016	0.261	−2.841	12.936
**Dependent Variable**	**Physical Activity Estimated by Running Activity Per Local Population**				
Number of observations	8979		8979		
Adjusted R2	0.82		0.71		
Optimal bandwidth for spatial kernel (m)	N/A		720		
AIC	24,718		16,470		

NOTES: Coeff. = estimated coefficient, Std. Err. = Standard Errors, and AIC = Akaike Information Criterion. *** = significant at 99% (*p* ≤ 0.01).

**Table 4:** Results from a global regression and GWR model of real estate price.

	Global Regression		Geographically Weighted Regression		
Independent Variable	Coeff.	Std. Err.	Mean	Min	Max
Building Age ***	0.045	0.009	−0.053	−5.521	2.091
Total Buildings ***	0.129	0.009	0.262	−0.216	2.228
Total Units ***	−0.091	0.008	−0.087	−1.279	0.352
Distance to City Center ***	−0.148	0.022	−1.003	−8.454	6.377
Avg. Commute (to Work) ***	−0.360	0.017	0.168	−1.382	3.687
Avg. Commute (to Home) ***	0.062	0.014	−0.127	−5.576	0.584
Vibrancy-Population ***	0.248	0.020	0.007	−7.508	16.005
Physical Activity ***	0.085	0.008	0.026	−0.608	12.405
Vibrancy-Residential *	−0.041	0.014	−0.097	−4.476	1.075
Vibrancy-Business **	−0.026	0.010	−0.016	−0.692	0.632
Vibrancy-Facility ***	0.103	0.015	−0.036	−1.044	1.628
Vibrancy-Quality *	−0.022	0.011	0.053	−0.920	1.759
**Dependent Variable**	***ln* (Apartment Compound Average Price)**				
Number of observations	6314		6314		
Adjusted R2	0.60		0.82		
Optimal bandwidth for spatial kernel (m)	N/A		1435.75		
AIC	12,454		8188		

NOTES: Coeff. = estimated coefficient, Std. Err. = Standard Errors, and AIC = Akaike Information Criterion. *** = significant at 99% (*p* ≤ 0.01); ** = significant at 95% (*p* ≤ 0.05); * = significant at 90% (*p* ≤ 0.10).
